# Zfp423 Binds Autoregulatory Sites in P19 Cell Culture Model

**DOI:** 10.1371/journal.pone.0066514

**Published:** 2013-06-06

**Authors:** Young-Wook Cho, Chen-Jei Hong, Aiju Hou, Peter M. Gent, Kuixing Zhang, Kyoung-Jae Won, Bruce A. Hamilton

**Affiliations:** 1 Departments of Medicine and Cellular and Molecular Medicine, Institute for Genomic Medicine and Moores UCSD Cancer Center, University of California San Diego School of Medicine, La Jolla, California, United States of America; 2 Department of Genetics, Institute for Diabetes, Obesity and Metabolism, School of Medicine, University of Pennsylvania, Philadelphia, Pennsylvania, United States of America; Université Paris-Diderot, France

## Abstract

Zfp423 is a 30 zinc finger transcription factor that forms regulatory complexes with EBF family members and factors targeted by canonical signaling pathways. *Zfp423* mutations produce a range of developmental abnormalities in mice and humans related to the ciliopathies. Surprisingly, computational analysis of clustered Zfp423 and partner motifs in conserved genomic sequences predicts enrichment in *Zfp423* and *Ebf* genes. In cell culture models selected for Zfp423 and EBF expression, we identify strong and reproducible occupancy of two *Zfp423* intronic sites using chromatin immunoprecipitation with multiple independent antibodies. Both sites are significantly enriched in either quantitative PCR or massively parallel sequencing assays. A site in intron 5 acts as a classical enhancer in transient assays, but does not require the consensus motif for activity, suggesting a redundant or modulatory role for Zfp423 binding in this context. We speculate that Zfp423 may repress this enhancer as part of a developmental ratchet.

## Introduction

Mammalian zinc finger protein 423 (mouse Zfp423, human ZNF423) is a transcriptional regulator important to development and disease. Mutations of *Zfp423* in mice produce severe midline defects in developing brain, most notably loss of the cerebellar vermis [1]–[3], as well as abnormalities in olfactory neurons [4] and brown fat [5]. The severity of these defects is highly variable and influenced by both modifier genes and non-genetic factors [6]. Germline mutations in human *ZNF423* result in a range of nephronophthisis-related ciliopathy (NPHP-RC) phenotypes, including characteristic defects in cerebellar vermis and kidney, with cellular deficits in DNA damage response [7]. ZNF423 may also play a role in human cancers. Epigenetic loss or reduction of ZNF423 expression in human neuroblastoma corresponds with lower response to retinoic acid therapy [8] and ectopic activation of Zfp423 in bone marrow cells induced B-cell leukemia in a mouse model [9].

Zfp423 is composed of 30 C2H2 zinc fingers, clustered into multi-finger domains reported to bind DNA or other transcription factors. Zfp423 (also known as ROAZ, OAZ, or Ebfaz) was first identified as a binding partner that inhibits Early B-cell factor (EBF, also known as Olf1) subfamily of basic helix-loop-helix transcription factors through its last three zinc fingers [10], [11]. Subsequent studies from a variety of contexts have identified additional interactions with transcription factors, including BMP-activated SMAD proteins [12], retinoic acid receptor RARβ [8], Notch intracellular domain [13], and DNA damage response related proteins, including poly (ADP-ribose) polymerase PARP1 [14] centrosomal protein CEP290 [15]. Several of these interactions are mutually inhibitory [12], [13]. Zfp423 has been proposed to regulate several target genes dependent on specific binding partners with their own DNA binding domains. Whether direct DNA binding by Zfp423 is required at the majority of these sites is not known.

In order to identify direct targets of Zfp423, we initiated an in silico strategy based on cross-species conservation of clustered consensus motifs [16] to predict candidate target sites. We used chromatin immunoprecipitation (ChIP), quantitative PCR (qPCR) and massively parallel sequencing to test occupancy of predicted sites in a standard cell culture model. Surprisingly, we found enrichment of consensus sites in or near genes encoding Zfp423, its paralog Zfp521, and two of four Ebf genes. Each of two sites identified within the *Zfp423* gene itself showed reproducible occupancy by Zfp423 in ChIP-PCR and ChIP-seq assays. The stronger of these sites, in *Zfp423* intron 5, also showed enhancer activity in heterologous classical promoter-reporter assays in P19 cells. Surprisingly, Zfp423 appears to act as a negative regulator at the stronger of the two sites, suggesting a negative feedback cycle that may be conditional on signaling and cell state.

## Results

### Conserved Zfp423-complex Binding Motifs are Enriched at *Zfp423* and *Ebf* Genes

**Figure 1:**
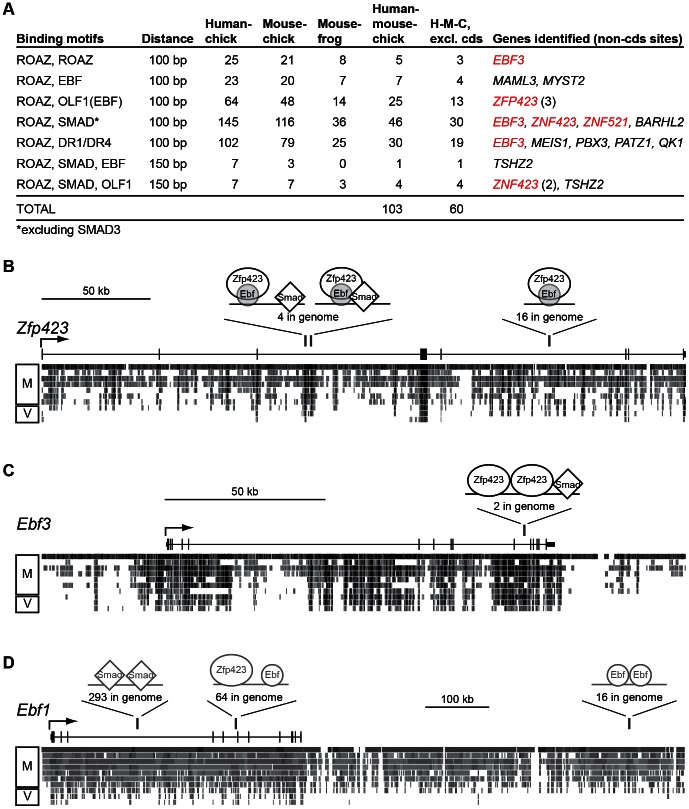
Conserved non-coding sequences suggest Zfp423 autoregulatory sites. Analysis of vertebrate conserved non-coding sequences for co-occurrence of consensus motifs for Zfp423 (ROAZ), Ebf family members (OLF1), BMP-responsive SMADs and nuclear receptor motifs recognized by retinoic acid receptors (DR1, DR4) shows unexpected enrichment in *Zfp423* and *Ebf* genes. (*A*) Table shows number of conserved sties for pairwise combinations of consensus binding sequences within 100 bp for two sites, 150 bp for three-site combinations. Alignments based on public assemblies of human (hg18), mouse (mm9), chicken (galGal3) and frog (xenTro2) genomes as of 5/1/2012. Number of sites conserved among the three species used for sorting is further reduced for sites excluding known protein coding sequences (H-M-C excl. cds). (*B*) Conserved, clustered sites identified in *Zfp423* include three positions where Zfp423 and Ebf matrix predictions overlap, are indicated by vertical above a diagram of exon structure and pairwise conservation between mouse and other mammal (M) and non-mammal vertebrate (V) species, showing the locations of predicted binding sites. Adapted from UCSC Genome Browser. Similar schematics for (*C*) *Ebf3* using the same parameters and (*D*) *Ebf1* using larger window sizes. Numbers indicate the frequency of similar predicted sites in the genome for the parameters used to identify each.

To identify candidate target genes for Zfp423, we first looked for consensus binding sites in regions conserved among vertebrate genomes (Figure 1). Using the web-based SynoR software tool, which uses a matrix representation to account for degeneracy in binding sites [16], we separately examined paired or clustered sites for Zfp423 [11] and its best-characterized binding partners, Ebf (including Olf1, represented by a distinct sequence matrix [17]), SMAD, and Retinoic acid receptor in sequences conserved across vertebrate species pairs (human-chick, mouse-chick, mouse-frog), across a range of parameters for number (1–4) of sites and maximum distance (100–400 bp) between sites for at least two component factors. Multiple binding matrices were used for Ebf (EBF_Q6 and OLF1_01) and SMAD (SMAD_Q6, SMAD_Q6_01, and SMAD4_Q6) family members. This analysis resulted in a surprisingly small number of sites genome wide; distributions of such sites for 100 bp windows with 2 sites or 150 bp windows for three sites are tabulated (Figure 1A). We identified 60 conserved non-coding sites containing a Zfp423 consensus site within 100 bp of either a consensus motif for one of its known binding partners or a second Zfp423 site, with syntenic site predictions in human, mouse and chicken. Surprisingly, four of these 60 robustly predicted clusters occur in the *Zfp423* and *Ebf3* genes (Figure 1B,C). Broadening the criteria to allow up to 200 bp between sites and to allow Ebf-only or SMAD-only clusters finds three additional sites in or adjacent to *Ebf1* (Figure 1D). This enrichment of clustered sites for known interacting factors in genes encoding those factors represents a dramatic enrichment above genome-wide expectation and led us to test whether these sites might be functional.

### Human IMR32 and Mouse P19 Cells Express Zfp423

**Figure 2:**
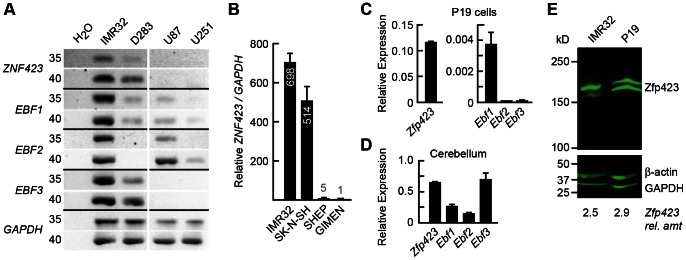
Cell culture models express Zfp423 and its binding partners. (*A*) Inverted gel images from semi-quantitative RT-PCR show expression of *ZNF423* and *EBF* family members in IMR32 (neuroblastoma), as well as D238 (medulloblastoma) cell lines. *ZNF423* was not detected from either U87 or U251 (glioblastoma) lines. Cycle numbers are indicated to the left. (*B*) Among four neuroblastoma cell lines, RT-qPCR shows high relative expression of *ZNF423* in IMR32 and SK-N-SH cells. Graph shows average and standard deviation for technical replicates of a single experiment. (*C*) Graph shows RT-qPCR expression values for *Zfp423* and *Ebf* RNAs in P19 cells, normalized to the geometric mean of *Gapdh*, *Pitpna*, and *Ppig* as reference genes. Error bars indicate range in technical replicates only. Analysis of an independent second culture showed similar results. (*D*) RT-qPCR expression values from postnatal day 3 mouse cerebellum as a primer control, normalized and scaled as in (*C*). Note significantly higher expression levels in tissue compared to P19. (*E*) Western blots show detection of Zfp423 in 25 µg total cellular protein from IMR32 or P19 cells, detected with a goat polyclonal antibody (E20) and visualized by infrared imaging. The doublet appearance is sometimes observed due to incomplete denaturation in the gel. Blot was re-probed with antibodies to β-actin and GAPDH as loading controls. Relative amounts of total Zfp423 reactivity compared to the loading controls are indicated.

Because Zfp423 expression is developmentally dynamic and complex with respect to endogenous cell types [1], [5], we briefly examined several cell lines that might be used as simplified model systems to assess direct binding to predicted targets sites with autoregulatory potential (Figure 2). Semi-quantitative RT-PCR from human cancer cell lines of neuroglial origin detected expression of *ZNF423* and at least two of three *EBF* genes in both neuroblastoma (IMR32) and medulloblastoma (D238) derived cell lines (Figure 2A). By contrast, *ZNF423* RNA was not detected in either of two glioblastoma lines (U87, U251). We further examined *ZNF423* expression among four neuroblastoma cell lines by quantitative RT-PCR (qRT-PCR). IMR32 and SK-N-SH each expressed high relative level of *ZNF423* RNA compared to SHEP or GIMEN in a single experiment (Figure 2B). As much of the biology of *ZNF423/Zfp423* has been studied in mice, we also examined a commonly used mouse teratocarcinoma cell line with neurogenic potential, P19, which was previously reported to express *Zfp423* and PARP [14]. Quantitative RT-PCR from P19 cells under standard growth conditions showed strong expression of *Zfp423*, some *Ebf1* and minimal, but detectable, expression of *Ebf2* and *Ebf3* (Figure 2C). All four RNAs were at substantially lower levels (both by absolute threshold cycle and relative to three reference genes) in P19 than in developing mouse whole cerebellum (Figure 2D), where *Zfp423* and all three *Ebf* members are readily detected. Accurate amplification was confirmed by melt curve analysis to ensure uniqueness of the amplified sequence and gel electrophoresis to confirm predicted size at endpoint. Similar quantitative results were obtained with two distinct primer sets for *Ebf3*. Western blot analysis confirmed expression of ZNF423/Zfp423 protein in IMR32 and P19 cells, relative to β-actin and GAPDH loading controls (Figure 2E). Zfp423 frequently appeared as a doublet under gel conditions that optimized its detection (see Methods); that both bands represented legitimate Zfp423 was supported both by their recognition with independent antibodies and by loss of both bands in extracts from either Zfp423-mutant tissues or cells treated with Zfp423-directed shRNA (see below). Based on expression of *ZNF423*/*Zfp423* and at least one *EBF*/*Ebf* member, IMR32 and P19 cells were selected for further experiments.

### Zfp423 Occupies Sites *Zfp423* Introns 3 and 5 in Mouse and Human Cells

**Figure 3:**
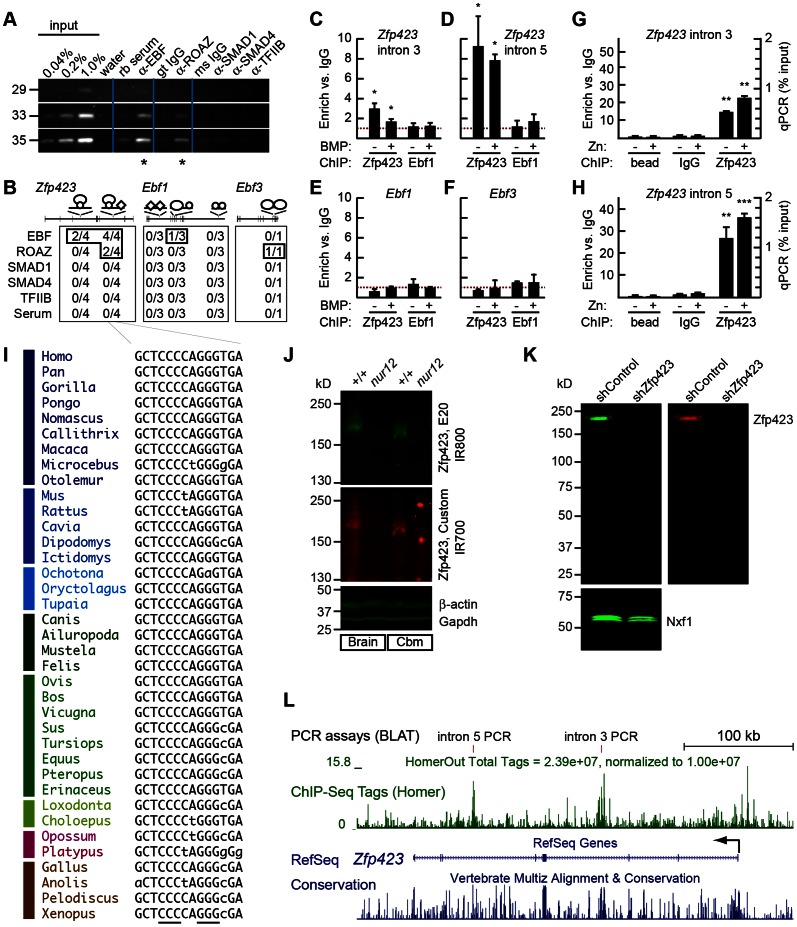
Zfp423 binds consensus sites in introns 3 and 5. (*A*) Semi-quantitative ChIP-PCR assays for the *ZNF423* intron 5 site in IMR32 cells, with commercial antibodies against the indicated factors compared with normal serum from same host species and titrated input chromatin. Cycle numbers are indicated to the left. (*B*) Frequency of observed enrichment for predicted sites tested in replicate experiments in IMR32 cells. Schematic indicates predicted binding sites for Zfp423 (oval), Ebf (circle) and SMAD (diamond). (*C*–*F*) Fold enrichment at the orthologous sites in mouse P19 cells, before or after 4 hour treatment with 200 ng/ml BMP2, measured by ChIP-qPCR. Data from Zfp423 antibody E20 are shown. (*C*) *Zfp423* intron 3, (*D*) *Zfp423* intron 5, (*E*) *Ebf1*, (*F*) *Ebf3*. (*G–H*) ChIP-qPCR using a custom, affinity-purified antibody against ZNF423 fusion protein shows higher fold discrimination at *Zfp423* sites in P19 cells. Experiments in A–B, C–F and G–H were performed independently by different investigators among the authors. * p≥0.05, ** p≥0.01, *** p≥0.001, t-test for comparison to IgG control for same condition and primer pair. (*I*) Alignment of the predicted binding site in intron 5 and syntenic sites from the indicated species shows strong sequence constraint that fits the overlapping Zfp423 (ROAZ) and EBF (OLF1) consensus motifs. (*J*) Western blot of mouse forebrain (Brain) and cerebellum (Cbm) extracts from wild-type littermate (+/+) or *Zfp423* null mutant (*nur12*) mice. The same blot was stripped and re-probed, using species-specific secondary antibodies coupled to alternate infrared fluors. Reactivity for β-actin and Gapdh are shown as internal loading controls. (*K*) Western blot of nuclear extracts from P19 cells treated with the indicated shRNA shows high degree of specificity for each Zfp423 antibody. Nxf1 is used as a loading control. Normalized shZfp423 signal <1% of control for each panel. (*L*) Screen shot from custom UCSC browser tracks showing normalized read density for ChIP-Seq from P19 cells, using custom ZNF423 antibody. Prominent peaks occur over the predicted sites in introns 3 and 5.

To test whether predicted sites are occupied in cells with relatively high levels of the indicated factors, we performed a series of chromatin immunoprecipitation (ChIP) experiments (Figure 3). Semi-quantitative PCR after ChIP detected ZNF423 binding above background in IMR32 cells at the intron 5 site in two experiments among four replicates (Figure 3A,B). A single experiment further suggested possible binding to a predicted site in *EBF3* gene. Interestingly, parallel ChIP-PCR experiments from the same lysates also detected EBF binding to several predicted sites, using an antibody with low discrimination among paralogous EBF members, though ChIP with several SMAD antibodies failed to detect binding under these conditions (Figure 3B).

To extend these results, we examined whether the mouse homologs bound to the predicted intronic sites of *Zfp423* and *Ebf* genes in P19 cells. Quantitative PCR detected modest but significant ChIP enrichment at the more 5′ (and more deeply conserved) site in intron 3 (Figure 3C) and much stronger enrichment at the intron 5 site (Figure 3D) compared to IgG controls. These experiments did not confirm significant binding of either Zfp423 or Ebf to the tested sites in *Ebf1* and *Ebf3*, nor did any site show a significant change in Zfp423 enrichment after activation of the SMAD pathway by BMP2 treatment (Figure 3E,F) in this cell type. To further confirm the binding of Zfp423 protein to the *Zfp423* intron sites, we developed an independent affinity-purified serum against conserved epitopes near the amino-terminus and optimized ChIP conditions for Zfp423 in mouse P19 cells (Figure 3G,H). Affinity-purified serum provided substantially higher discrimination and higher percentage of input recovered compared to commercial preparations. Addition of 10 mM Zinc to each step of chromatin purification added a nominal increase in recovery. These experiments replicated the strong enrichment of the intron 5 site and provided additional evidence for binding at the intron 3 site, although the intron 5 site showed greater enrichment with both antibodies. Alignment of the predicted intron 5 binding site among orthologues showed that the consensus motif–especially the core CCCnnGGG–was strongly conserved among sequenced vertebrate genomes (Figure 3I). Western blots of total protein extracts from wild-type and *Zfp423^nur12^* null mutant mouse forebrain and cerebellum showed specificity of both antibodies, with complete loss of Zfp423 in mutant brain (Figure 3J). The same membrane was probed sequentially with each antibody, imaged to ensure complete stripping in between, and imaged in alternate channels for the two species-specific secondary antibodies. As the custom serum gave stronger ChIP signal, its Western blot signal was imaged in the lower-sensitivity channel. Subsequent probing with antibodies against mouse β-actin and Gapdh confirmed similar levels of protein loaded across samples. To further test the specificity of antibodies in our ChIP experiments, we performed Western blots on nuclear extracts of P19 cells expressing either shRNA directed against Zfp423 or a control (Figure 3K). The E20 antibody had a very faint cross-reacting band at ∼65 kD not seen with the custom serum, while the custom serum had a very faint cross-reacting band at ∼48 kD not seen with E20, underscoring the value of using independent antibodies to confirm ChIP signals. An antibody against the mRNA nuclear export factor Nxf1 [18] was used as a loading control.

To confirm Zfp423 occupancy of these predicted sites with a different assay measure, we used the custom antibody for a pilot genome-wide ChIP-Seq experiment (Figure 3L). Zfp423 ChIP-enriched DNA was subjected to massively parallel sequencing on the Illumina GA-II platform, generating 23,894,656 reads mapped to the mm9 assembly of the mouse reference genome using Bowtie and allowing maximum 2 mismatches. Peaks were called in Homer based on Poisson p-value over local region (<10–4), fold enrichment over local region (>4), and FDR (<0.001). While the singleton nature of this design and potential for remaining background limited the genome-wide confidence for discovery of potential new targets in this one-sample data set, all three predicted sites in *Zfp423* introns 3 and 5 were called as peaks and enriched relative to other sites in a 400 kb window encompassing the *Zfp423* gene, providing further evidence for the prior hypothesis of selective binding by Zfp423 at these sites and for the potential of a conserved autoregulatory circuit at *Zfp423*. The intron 5 site was among the top 30 scores on chromosome 8 using the peak-finding algorithm in Homer. Moreover, the peak was composed of two adjacent, facing sets of forward and reverse strand reads, with the modes for each strand separated by approximately the model distance of the sheared chromatin used for constructing the sequencing library.

### *Zfp423* Intron 5 Fragment has Enhancer Activity in P19 Cells

**Figure 4:**
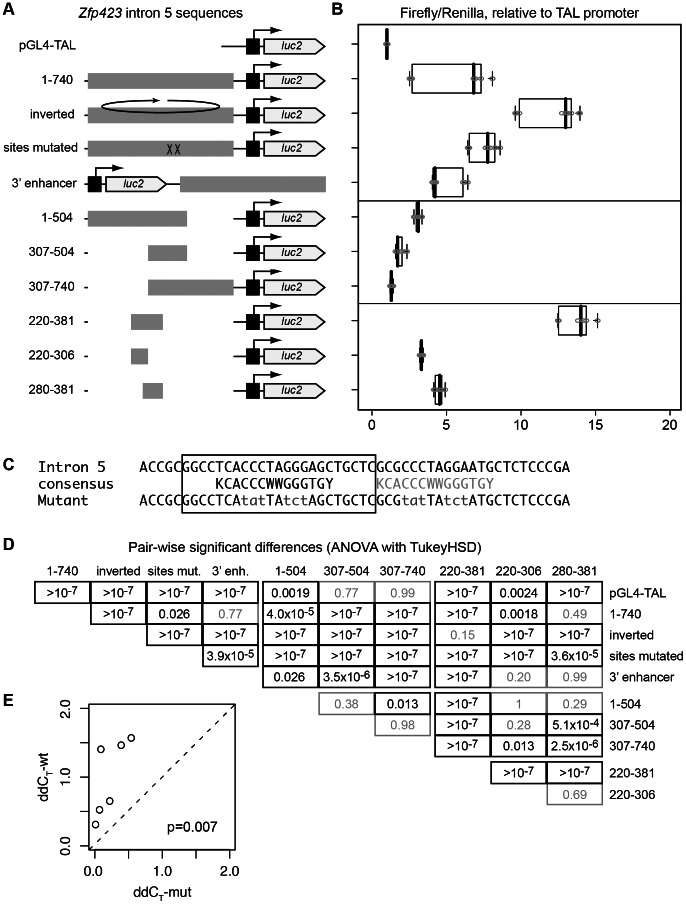
*Zfp423* intron 5 binding site is an enhancer. (*A*) Reporter constructs are shown schematically. A pTAL minimal promoter was inserted upstream of the firefly luciferase reporter in pGL4. Fragments of the *Zfp423* intron 5 putative enhancer were placed as indicated by the alignment of grey boxes. Position of the Zfp423 consensus match is indicated by XX in the “sites mutated” construct. Horizontal lines indicated constructs tested together, with pGL4-TAL repeated in each group. (*B*) Reporter gene expression levels, expressed as the ratio of firefly luciferase to co-transfected Renilla luciferase enzymatic activity measured by luminescence. Each measurement was normalized to the average of control vector (pGL4-TAL) samples run on the same day. For each construct, 2–3 DNA preparations were each used in 2–4 independent co-transfections for 9–12 (top eight constructs) or 6 (bottom three constructs) measurements. (*C*) Sequence changes in the sites mutated construct. Top line shows sequence of the mouse reference clone. The overlapping ROAZ (Zfp423), OLF1 site predicted by SynoR is boxed. Consensus Zfp423 (ROAZ) binding site and an adjoining consensus half-site (grey text) are indicated. Bottom line shows mutated sequence, with altered residues in grey lowercase. (*D*) As one estimate of the significance level for apparent shifts between constructs, normalized expression values were compared by ANOVA, followed by Tukey's Honest Significant Differences pair-wise comparisons. Calculated p-values are indicated in the intersection between construct designations. Potential scaling differences between sequential experiments on different days (indicated by gaps between cells in the table) may violate some assumptions of the test. (*E*) ChIP-qPCR performed for transfected plasmids shows a higher enrichment index compared to input and IgG controls (calculated as ΔΔC_T_ for carrying the wild-type site than for the mutated sites shown in (C). The pairwise comparison was significant at p = 0.007 by t-test or 0.03 by the nonparametric equivalent (Wilcoxon signed rank test).

To test whether the strongly-bound site in intron 5 has enhancer activity, we cloned it and several derivatives into a plasmid with a basal promoter driving firefly luciferase as a reporter, co-transfected each construct with a single Renilla luciferase reporter as a transfection control into P19 cells, and measured the ratio of luciferase activities for replicate transfections with each of three independent DNA preparations for each intron 5 construct (Figure 4). The base construct included a 740 bp fragment fully encompassing the vertebrate-conserved sequence, in the same orientation relative to the direction of transcription as it occurs in *Zfp423* (Figure 4A). This showed substantial enhancer activity relative to the base vector, pGL4-TAL (Figure 4B). Shorter fragments derived from the 740 bp sequence also showed activity, including a fragment with the overlapping Zfp423/Ebf predicted binding sites mutated (Figure 4C). Having independent DNA preparations with substantial replication measures for each tested construct allowed us to compare relative enhancer strengths compared to the pGL4-TAL base vector transfected on the same day for each experiment with a robust statistical comparison (Figure 4D). Placing the full 740 bp sequence in the reverse orientation showed even higher activity in this assay, while placing it 3′ to the reporter showed essentially the same activity level as the initial construct, satisfying the classical criteria for a transcriptional enhancer. Surprisingly, mutation of several nucleotides in the putative Zfp423 recognition sites to destroy the consensus motifs (location indicated by XX in Figure 4A) did not diminish, but rather slightly increased expression, suggesting that direct binding by Zfp423 may not be self-activating, but perhaps act as negative regulators in some context (the increase was statistically significant by ANOVA; see comparison of ‘sites mut.’ to ‘1–740’ in Figure 4D). As an indicator of direct binding, we quantified relative ChIP/input ratios by qPCR for transfected plasmids (Figure 4E). Six of six paired comparisons (duplicate transfection of three independent preparations of each plasmid) showed greater enrichment index for wild-type than mutated sequence (p = 0.007, paired t-test). A series of deletion constructs suggested the presence of both positive and negative elements within the enhancer and a minimal element of 162 bp that omits the Zfp423 consensus sites had the highest activation level of all tested fragments. In addition to confirming the intron 5 site as an enhancer, these data suggested that Zfp423 binding might be a negative regulator of its own transcription.

### Zfp423 Overexpression Represses the *Zfp423* Intron 5′ Enhancer in P19 Cells

**Figure 5:**
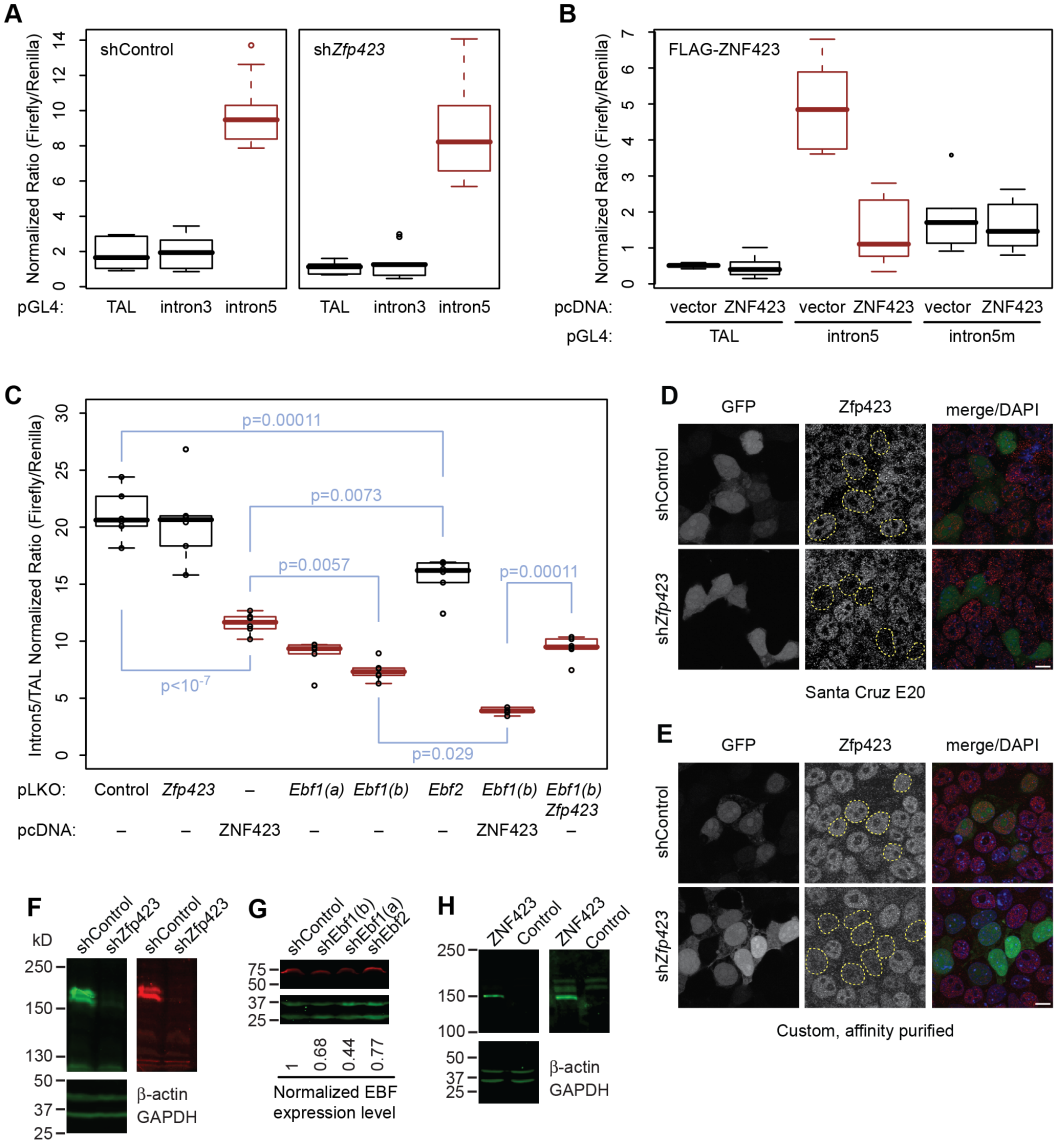
*Zfp423* overexpression represses intron 5 enhancer activity in P19 cells. (*A*) pGL4 reporter with the intron 5 enhancer was similarly active when co-transfected with shRNA directed against *Zfp423* or a control. A similar plasmid with a region encompassing the intron 3 binding site had no activity above the pTAL minimal promoter. (*B*) Co-transfection with a plasmid expressing FLAG-tagged human ZNF423 reduced expression of the intron 5 reporter relative to a pcDNA vector control. This effect did not occur between paired samples with the Zfp423 consensus motifs mutated (intron5m). (*C*) An independent series of co-transfection assays indicates Ebf1-dependence of the intron 5 enhancer in P19 cells. ZNF423 overexpression and *Ebf1* knockdown shows comparable reductions in enhancer activity (p<10^−7^, Tukey HSD pair-wise comparisons to control after ANOVA). Combining ZNF423 overexpression and *Ebf1* knockdown showed further reduction in reporter activity (p = 0.03–0.0000001), while shRNA against endogenous *Zfp423* showed no additional effect on activity in *Ebf1* knockdown cells. Each construct was assayed in duplicate for each of three independent DNA preparations. (*D–E*) Immunofluorescent detection of Zfp423 by either E20 (*D*) or our custom affinity purified antibody (*E*) in P19 cells transfected with plasmid expressing EGFP and either scrambled control or Zfp423-targeted shRNA shows specific reduction or loss of immunoreactivity in nuclei of Zfp423 depleted cells (outline in middle panels). (*F*) Western blotting with either commercial (left panel) or custom (right panel) antibody showed effective reduction of P19 Zfp423 levels after transfection with plasmid expressing *Zfp423*-directed shRNA compared with internal loading controls. Repeated experiments showed 60–99% reduction. (*G*) Western blotting with a pan-EBF antibody showed moderate reduction (44–68% of control levels) after transfection with plasmid expressing *Ebf1*-directed shRNA. (*H*) Western blotting showed overexpression of ZNF423 in pcDNA-FLAG-ZNF423 transfected cells. The transfected product showed 1.4× endogenous level, indicating 2.4-fold expression level for endogenous and transfected products combined.

Since the predicted Zfp423 binding sites were not required for enhancer activity on a heterologous promoter and deletion of these binding sites appears to increase enhancer strength, we next tested whether ZNF423 expression had any effect on reporter gene expression (Figure 5). Co-transfection of reporter constructs with a short hairpin RNA (shRNA) targeting *Zfp423* mRNA that reduces Zfp423 to ∼20% normal levels in P19 cells did not reproducibly alter reporter activity in triplicate measures from a single DNA preparation per construct (Figure 5A). In parallel, a construct including the conserved segment at the intron 3 site showed no enhancer activity either before or after knockdown of *Zfp423* RNA, in comparison to pGL4-TAL. However, overexpression of human ZNF423 substantially attenuated expression of the reporter, compared to pcDNA expression vector control (Figure 5B), providing further evidence for a negative effect of Zfp423 at high expression levels. This effect was not seen in co-transfection when the Zfp423 binding sites were mutated, suggesting a direct effect of binding. Since the Zfp423 binding site overlaps a predicted binding site for EBF family members, we then tested whether reducing Ebf expression in P19 cells affects reporter activity (Figure 5C). From duplicate measurements for each of three independent DNA preparations per construct, we again saw no effect of shRNA targeting *Zfp423*, but a highly significant decrease on overexpression of human ZNF423 (p<10^−7^, Tukey HSD pair-wise test after one-factor ANOVA). Targeting *Ebf1* with shRNA resulted in a similar loss of reporter expression (p<10^−7^), suggesting that Ebf1 contributes to activation of this enhancer. Targeting *Ebf2*, which appeared to be expressed at lower levels based on qRT-PCR data (Figure 2C) had only a modest effect. Simultaneously targeting both *Ebf1* and *Zfp423* by co-transfection of shRNA constructs was not significantly different from targeting *Ebf1* alone. However, overexpression of ZNF423 together with *Ebf1* shRNA further reduced reporter expression (p = 0.029) below the level achieved with *Ebf1* shRNA alone. Expression of the GFP-marked shRNA construct dramatically reduced Zfp423 immunofluorescence in P19 nuclei using either the E20 (Figure 5D) or custom made (Figure 5E) antibody. Western blots confirmed effective reduction of protein expression after shRNA transfections (Figure 5F,G) and overexpression after transfection with human ZNF423 expression plasmid (Figure 5H).

## Discussion

Zfp423 acts coordinately with several lineage and signaling-dependent transcription complexes that are important for precursor cell differentiation, including direct interactions with Ebf, BMP-dependent Smad, retinoic acid receptor, and Notch intracellular domain proteins [5], [8], [10], [12], [13]. While dynamically expressed in embryos, postnatal Zfp423 expression attenuates with maturation in many cell lineages and artificially sustained expression in at least the olfactory lineage inhibits normal maturation [4], [10], [19]. Early observations in the olfactory system by Reed and co-workers indicated that Zfp423 is co-expressed with Ebf1, which Zfp423 inhibits, in immature cells. As these precursors mature, Ebf1 stays on while Zfp423 turns off, promoting Ebf1 transactivation of lineage-specific target genes during differentiation [10]. Conversely, re-expression of Zfp423 reverts the expression of several key genes to an immature state [4]. How this stereotyped progression is regulated is not known, but the sequence of expression states would be consistent with a self-regulating transcriptional network. Our results demonstrate that endogenous Zfp423 directly binds two conserved non-coding sites in its own gene, providing a potential autoregulatory mechanism, in both mouse and human cell culture models; that the intron 5 site acts as an enhancer by classical reporter assays in a P19 cell culture model; and that while this enhancer does not require Zfp423 or its predicted binding site for activity, the enhancer is substantially suppressed by high level expression of ZNF423. These results provide a plausible mechanism for signal-induced progression of precursor cells dependent on Zfp423, using autoregulatory sites to scaffold feed-forward or feedback loops, depending upon the cell state, signaling environment, and the state of co-regulatory factors.

The intron 5 enhancer element is functionally blocked by overexpression of ZNF423. This may suggest an autoregulatory negative feedback loop under conditions of high Zfp423 expression, even though knockdown of endogenous *Zfp423* RNA (to ∼24% of control levels) does not appear to alter enhancer activity. It is possible that the repression caused by exogenous expression is indirect and a consequence of ZNF423 activity on other genes. However, as the enhancer is directly bound by Zfp423 in a conserved fashion and mutation of the presumed binding sites eliminates the effect of overexpression (Figure 5), this seems to us less likely. Alternatively, it is possible that in this experimental system and under these conditions, the level of enhancer occupancy (or its context for other binding partners) is not sensitive to reduced levels, while addition of exogenous ZNF423 alters either the percent occupancy or composition of the binding complex sufficiently to repress activation. For example, while knockdown of Ebf1 reduced reporter expression, simultaneous knockdown of Ebf1 and Zfp423 was somewhat less effective. It is notable that the cell culture model used for these extensive enhancer activity studies, P19, expresses lower levels of *Zfp423* RNA than developing neural tissues, suggesting that the response we see to ∼2.4X higher levels of ZNF423 in the culture model (Figure 5H) may indeed be relevant to levels achieved in developing cells. We propose that this site might play a role in either limiting Zfp423 accumulation or, more provocatively, providing a developmental ratchet in which Zfp423 alone or in progressive complexes with one or more binding partners serves to turn off its own expression, to allow the cell to exit an immature cell state and facilitate developmental progression.

Our results also provide some information on predictive algorithms for transcription factor binding. While many of the sites we examined do not appear occupied under the narrow conditions tested, we do find compelling evidence for binding at several sites, and particularly strong evidence for binding in both mouse and human at a clearly functional site in intron 5. Regardless of whether the other predicted sites are occupied under conditions or not, the predictive approach need not be perfect to be a useful guide for early experiments where legitimate targets are not defined. In this example, the occurrence of clustered motifs for transcription factors known to interact in a complex facilitated the identification of an apparent autoregulatory site for a key transcription factor important for the development of both mice and humans. All together, our results strongly support both Zfp423 occupancy and functional enhancer activity for at least one predicted conserved segment. As most enhancers are cell-type specific [20], further analysis of genome-wide binding in a wider variety of cellular contexts will be required to test the generality of such predictions.

## Materials and Methods

### Antibodies

Zfp423 antibodies E20 and D16 were obtained from Santa Cruz Biotechnology (Figure 3A–F). Additional custom antisera were raised in rabbit against His-fusion protein expressing either residues 1–180 or 247–407 relative to human ZNF423 reference sequence NP_055884.2 and affinity-purified against the immunogen. ChIP experiments reported here used serum against 247–407 (Figure 3G–I); serum against residues 1–180 performed less robustly in ChIP assays and was not considered further. EBF antibodies were a gift from Dr. Randall Reed (Figure 3A,B) or purchased from Santa Cruz Biotechnology (H300, Figure 3C–F). SMAD antibodies A4 and H552 were obtained from Santa Cruz Biotechnology. Western blots were developed with infrared-conjugated secondary antibodies (Rockland), detected on a Li-Cor Odyssey Imaging Station, and quantified in the ImageJ software package. Monoclonal antibodies against β-actin (AC-74, Sigma) and GAPDH (GT239, GeneTex) were used to verify equal loading by detection of internal standards.

### Cell Lines

Neuroblastoma IMR32 [21] was originally obtained from ATCC [22] and passaged in the authors' laboratory to obtain a more adherent phenotype for ChIP. Medulloblastoma line D238 [23] was obtained from ATCC. Cell lines or cDNA from and glioblastoma U87 and U251 [24], [25] were obtained from Dr. Frank Furnari. Mouse P19 cells [26] were obtained from the laboratory of Dr. Michael G. Rosenfeld.

### Chromatin Immunoprecipitation

Cells were crosslinked in 1% formaldehyde, sonicated, and subjected to standard ChIP purification with the indicated antibodies at ∼2 µg per 1×10^7^cells. For ChIP-Seq, sheared chromatin was treated essentially as described [27] and converted to sequencing library for massively parallel sequencing on the Illumina GA-II platform. Sequencing was carried out in the UCSD BIOGEM Core facility. Analysis of resulting sequence reads performed in the Homer package [27] identified 13,765 Znf423 peaks at a calculated false discovery rate <0.001. Tags were normalized to number of mapped reads for visualization in the UCSC Genome Browser as a custom track.

### Quantitative PCR

PCR primers (Supplemental Table S1) were designed using Primer3 online tool [28]. Real-time PCR amplification was quantified by stimulated fluorescence of SYBR green dye on a Bio-Rad CFX-96 instrument. Relative quantification of *ZNF423* among neuroblastoma lines compared expression in each sample to *GAPDH* as a conventional control by the ΔΔCt method. For quantitative RT-PCR from mouse tissue and P19 cells, values were normalized to the geometric means of *Gapdh*, *Pitpna*, and *Ppig* reference genes and expressed as 2^−Ct(gene)/Ct(reference)^. Quantitative PCR from ChIP samples used either a pre-immune IgG mock ChIP or input fraction as indicated for relative quantification among samples.

### Western blots

Homogenized tissue, whole cell, or nuclear protein extracts were prepared in ice-cold RIPA buffer with protease inhibitor cocktail (Sigma), 10 mM DTT, 10 mM sodium orthovanadate, 8 M urea and treated with 100 U Benzonase nuclease (EMD) until minimize viscosity. Extracts were incubated in a sample buffer (50 mM Tris pH 6.8, 2% SDS, 0.1% Bromophenol blue 10% Glycerol, 33 mM DTT, 0.1 M β-mercaptoethanol, 8 M urea at room temperature for 10 minutes prior to loading, as boiling was found to dramatically reduce signal strength. After gel electrophoresis, proteins were transferred to PVDF membrane (Immobilion-FL). Membranes were incubated with the indicated primary antibodies (1∶500) in Odyssey blocking buffer (Li-Cor) with 0.2% Tween 20. Immunoreactivity was measured with infrared-conjugated secondary antibodies (Rockland) detected on an Odyssey imaging station (Li-Cor).

### Luciferase Assays

Plasmid pGL4 including the pTAL minimal promoter was modified to incorporate the indicated fragments from mouse *Zfp423* sites and sequence verified. For luciferase reporter assays, P19 cells were co-transfected in triplicate for each experiment and the ratio of firefly luciferase to *Renilla* luciferase activity was taken as the experimental measure. Experiments were replicated 3 times with independent DNA preparations.

### Statistical Tests

Standard one and two-sample t-tests were performed in the GraphPad online calculator (http://www.graphpad.com/quickcalcs/) or in R; all other statistical tests were performed in the R version 2.8.1 (2008-12-22) base package environment.

## Supporting Information

Table S1 **Primer sequences.** The amplification target, primer sequences, predicted product size and locations of resulting data in the published figures is indicated for each PCR assay. Primers used to verify sequence of the ZNF423 cDNA clone are numbered sequentially.
(DOC)

## References

[1] Alcaraz WA, Gold DA, Raponi E, Gent PM, Concepcion D, et al (2006) Zfp423 controls proliferation and differentiation of neural precursors in cerebellar vermis formation. Proc Natl Acad Sci U S A 103: 19424–19429. 17151198 10.1073/pnas.0609184103 PMC1748242

[2] Cheng LE, Zhang J, Reed RR (2007) The transcription factor Zfp423/OAZ is required for cerebellar development and CNS midline patterning. Dev Biol 307: 43–52. 17524391 10.1016/j.ydbio.2007.04.005 PMC2866529

[3] Warming S, Rachel RA, Jenkins NA, Copeland NG (2006) Zfp423 is required for normal cerebellar development. Mol Cell Biol 26: 6913–6922. 16943432 10.1128/MCB.02255-05 PMC1592861

[4] Cheng LE, Reed RR (2007) Zfp423/OAZ participates in a developmental switch during olfactory neurogenesis. Neuron 54: 547–557. 17521568 10.1016/j.neuron.2007.04.029 PMC2866517

[5] Gupta RK, Arany Z, Seale P, Mepani RJ, Ye L, et al (2010) Transcriptional control of preadipocyte determination by Zfp423. Nature 464: 619–623. 20200519 10.1038/nature08816 PMC2845731

[6] Alcaraz WA, Chen E, Valdes P, Kim E, Lo YH, et al (2011) Modifier genes and non-genetic factors reshape anatomical deficits in Zfp423-deficient mice. Hum Mol Genet 20: 3822–3830. 21729880 10.1093/hmg/ddr300 PMC3168291

[7] Chaki M, Airik R, Ghosh AK, Giles RH, Chen R, et al (2012) Exome Capture Reveals ZNF423 and CEP164 Mutations, Linking Renal Ciliopathies to DNA Damage Response Signaling. Cell 150: 533–548. 22863007 10.1016/j.cell.2012.06.028 PMC3433835

[8] Huang S, Laoukili J, Epping MT, Koster J, Holzel M, et al (2009) ZNF423 is critically required for retinoic acid-induced differentiation and is a marker of neuroblastoma outcome. Cancer Cell 15: 328–340. 19345331 10.1016/j.ccr.2009.02.023 PMC2693316

[9] Miyazaki K, Yamasaki N, Oda H, Kuwata T, Kanno Y, et al.. (2009) Enhanced expression of p210BCR/ABL and aberrant expression of Zfp423/ZNF423 induce blast crisis of chronic myelogenous leukemia. Blood. 10.1182/blood-2007-05-088724 19234145

[10] Tsai RY, Reed RR (1997) Cloning and functional characterization of Roaz, a zinc finger protein that interacts with O/E-1 to regulate gene expression: implications for olfactory neuronal development. J Neurosci 17: 4159–4169. 9151733 10.1523/JNEUROSCI.17-11-04159.1997 PMC6573535

[11] Tsai RY, Reed RR (1998) Identification of DNA recognition sequences and protein interaction domains of the multiple-Zn-finger protein Roaz. Mol Cell Biol 18: 6447–6456. 9774661 10.1128/mcb.18.11.6447 PMC109231

[12] Hata A, Seoane J, Lagna G, Montalvo E, Hemmati-Brivanlou A, et al (2000) OAZ uses distinct DNA- and protein-binding zinc fingers in separate BMP-Smad and Olf signaling pathways. Cell 100: 229–240. 10660046 10.1016/s0092-8674(00)81561-5

[13] Masserdotti G, Badaloni A, Green YS, Croci L, Barili V, et al (2010) ZFP423 coordinates Notch and bone morphogenetic protein signaling, selectively up-regulating Hes5 gene expression. J Biol Chem 285: 30814–30824. 20547764 10.1074/jbc.M110.142869 PMC2945575

[14] Ku MC, Stewart S, Hata A (2003) Poly(ADP-ribose) polymerase 1 interacts with OAZ and regulates BMP-target genes. Biochem Biophys Res Commun 311: 702–707. 14623329 10.1016/j.bbrc.2003.10.053

[15] Chaki M, Airik R, Ghosh AK, Giles RH, Chen R, et al.. (2012) Exome capture reveals ZNF423 and CEP164 mutations, linking renal ciliopathies to DNA damage response signaling. Cell In Press. 10.1016/j.cell.2012.06.028 PMC3433835 22863007

[16] Ovcharenko I, Loots GG, Nobrega MA, Hardison RC, Miller W, et al (2005) Evolution and functional classification of vertebrate gene deserts. Genome Res 15: 137–145. 15590943 10.1101/gr.3015505 PMC540279

[17] Wang MM, Tsai RY, Schrader KA, Reed RR (1993) Genes encoding components of the olfactory signal transduction cascade contain a DNA binding site that may direct neuronal expression. Molecular and cellular biology 13: 5805–5813. 7689152 10.1128/mcb.13.9.5805-5813.1993 PMC360324

[18] Floyd JA, Gold DA, Concepcion D, Poon TH, Wang X, et al (2003) A natural allele of Nxf1 suppresses retrovirus insertional mutations. Nat Genet 35: 221–228. 14517553 10.1038/ng1247 PMC2756099

[19] Warming S, Suzuki T, Yamaguchi TP, Jenkins NA, Copeland NG (2004) Early B-cell factor-associated zinc-finger gene is a frequent target of retroviral integration in murine B-cell lymphomas. Oncogene 23: 2727–2731. 15048087 10.1038/sj.onc.1207452

[20] Heintzman ND, Hon GC, Hawkins RD, Kheradpour P, Stark A, et al (2009) Histone modifications at human enhancers reflect global cell-type-specific gene expression. Nature 459: 108–112. 19295514 10.1038/nature07829 PMC2910248

[21] Tumilowicz JJ, Nichols WW, Cholon JJ, Greene AE (1970) Definition of a continuous human cell line derived from neuroblastoma. Cancer Res 30: 2110–2118. 5459762

[22] Wen G, Wessel J, Zhou W, Ehret GB, Rao F, et al (2007) An ancestral variant of Secretogranin II confers regulation by PHOX2 transcription factors and association with hypertension. Hum Mol Genet 16: 1752–1764. 17584765 10.1093/hmg/ddm123 PMC2695823

[23] Friedman HS, Burger PC, Bigner SH, Trojanowski JQ, Wikstrand CJ, et al (1985) Establishment and characterization of the human medulloblastoma cell line and transplantable xenograft D283 Med. J Neuropathol Exp Neurol 44: 592–605. 4056828 10.1097/00005072-198511000-00005

[24] Ponten J, Macintyre EH (1968) Long term culture of normal and neoplastic human glia. Acta Pathol Microbiol Scand 74: 465–486. 4313504 10.1111/j.1699-0463.1968.tb03502.x

[25] Westermark B, Ponten J, Hugosson R (1973) Determinants for the establishment of permanent tissue culture lines from human gliomas. Acta Pathol Microbiol Scand A 81: 791–805. 4359449 10.1111/j.1699-0463.1973.tb03573.x

[26] McBurney MW, Rogers BJ (1982) Isolation of male embryonal carcinoma cells and their chromosome replication patterns. Dev Biol 89: 503–508. 7056443 10.1016/0012-1606(82)90338-4

[27] Heinz S, Benner C, Spann N, Bertolino E, Lin YC, et al (2010) Simple combinations of lineage-determining transcription factors prime cis-regulatory elements required for macrophage and B cell identities. Molecular cell 38: 576–589. 20513432 10.1016/j.molcel.2010.05.004 PMC2898526

[28] Rozen S, Skaletsky HJ (2000) Primer3 on the WWW for general users and for biologist programmers. In: Krawetz S, Misener S, Bioinformatics Methods and Protocols: Methods in Molecular Biology. Totowa, NJ: Humana Press. 365–386. 10.1385/1-59259-192-2:365 10547847

