# Supplementary material for: Zfp423 Binds Autoregulatory Sites in P19 Cell Culture Model
Source: PLoS One. 2013 Jun 6;8(6):e66514. doi: 10.1371/journal.pone.0066514 (PMC3675209; doi:10.1371/journal.pone.0066514)
Supplement: Table S1 — Primer sequences. The amplification target, primer sequences, predicted product size and locations of resulting data in the published figures is indicated for each PCR assay. Primers used to verify sequence of the ZNF423 cDNA clone are numbered sequentially. (DOC) [file pone.0066514.s001.doc]

Table S1: Summary of primer sequences

| **Location** | **Primer sequences** | **Product size (bp)** | **Figure** |
| --- | --- | --- | --- |
| ***RT-PCR*** |  |  |  |
| ZNF423 exons 7-8 | Forward: 5’-CCAAGCTCCTCTGTCACCTC-3’  Reverse: 5’-GATGTGCTGCTGCAACTTGT-3’ | 113 | 2A |
| ZNF423 exon 4 | Forward: 5’-AAGTCCTTCATCCGCTTGAGC-3’  Reverse: 5’-GTGGCAGTGATACTTCTTGTCG-3’ | 159 | 2B |
| Zfp423 exons 7-9 | Forward: 5’-CACAGTCTTCGTCCAGGCCA-3’  Reverse: 5’-CTCACTGTGCGTGCTGGCTCA-3’ | 152 | 2C,D |
| Ebf1 exons 2-3 | Forward: 5’-CGGAAATCCAACTTCTTCCA-3’  Reverse: 5’-GTCTTTTCGCTGTTGGCTTC-3’ | 125 | 2C,D |
| Ebf2 exons 3-5 | Forward: 5’-TGACAAAGAGCAAGGCAATG-3’  Reverse: 5’-TGGTGACAGAGTCGATGAGC-3’ | 122 | 2C,D |
| Ebf3 exons 2-3 | Forward: 5’-CGGAAATCCAATTTCTTCCA-3’  Reverse: 5’-TTCTCGTTGTTCGGCTCTTT-3’ | 122 | 2C,D |
| Gapdh ORF | Forward: 5’-AGAAACCTGCCAAGTATGAT-3’  Reverse: 5’-TGTCATACCAGGAAATGAGC-3’ | 198 | 2C,D |
| Pitpna exons 4-7 | Forward: 5’-ACTGCAGAACCGTTATTACT-3’  Reverse: 5’-GTTTATGCACATTCTCCTGG-3’ | 105 | 2C,D |
| Ppig exons 5-6 | Forward: 5’-GGGACAGGGAAATCAACTCA-3’  Reverse: 5’-CCTCCTCTTCCATTCCCTTC-3’ | 116 | 2C,D |
| ***ChIP-PCR*** |  |  |  |
| ZNF423 intron 3a | Forward: 5’-AAACACAATCACCTCCTCTG-3’  Reverse: 5’-TGGCAATTTCTCTCAGACACC-3’ | 149 | 3B |
| ZNF423 intron 3b | Forward: 5’-TGAAGGAAGCTCAAACACAC-3’  Reverse: 5’-CCAGATCCACACCAGGAAG-3’ | 117 | 3B |
| ZNF423 intron 5 | Forward: 5’-TGAGGCCACGGTAAATTCTG-3’  Reverse: 5’-GTTCAGCCAAGACAGCCAAC-3’ | 127 | 3A,B |
| EBF1 intron 6 | Forward: 5’-GTAAGTGTTTACAGATGGCAAATG-3’  Reverse: 5’-AAGGGCTTTCTCATGGAGTCTT-3’ | 161 | 3B |
| EBF1 intron 8 | Forward: 5’-CTCCCAAAGGTACTGCAAGG-3’  Reverse: 5’-CAGAGAAGCCCTCAAACAGG-3’ | 99 | 3B |
| EBF1 distal | Forward: 5’-TAGCAAAGCCACCTCCAAAG-3’  Reverse: 5’-TTTCACCAGCCTCACAACTC-3’ | 95 | 3B |
| EBF3 intron 11 | Forward: 5’-CTAATGAACCCGACTTTCTG-3’  Reverse: 5’- AGCATCCTTGACTGATTGG-3’ | 92 | 3B |
| Zfp423 intron 3 | Forward: 5’-GGAGCAGACAGGCACTTAGC-3’  Reverse: 5’-ACACCCAATTACCCCCTGAC-3’ | 108 | 3C |
| Zfp423 intron 5 | Forword:5’-CAGCAGAAGGGGCTGTTCTA-3’  Reverse:5’-GGGATTAGGCGGAATTTACC-3’ | 111 | 3D |
| Ebf1 intron 8 | Forward: 5’-ACGTGGCCTTTGATCAACTC-3’  Reverse: 5’-CTGCAAGGCACAAAATGCTA-3’ | 185 | 3E |
| Ebf3 intron 11 | Forward: 5’-GAACCCGACTTTCTGAGCTG-3’  Reverse: 5’-TCAAACACCATTCGGTGCTA-3’ | 173 | 3F |
| Zfp423 intron 3 | Forward: 5’- ATTTCTCTCAGACACCCAATTACC-3’  Reverse: 5’- AATTTAGTATCCAGCCGAAAACAC-3’ | 159 | 3G |
| Zfp423 intron 5 | Forward: 5’-TCATTTGTATGCAGAAGGGATTAG-3’  Reverse: 5’-AATGAAAATGATTTCGATGTTGG-3’ | 196 | 3H |
| ***Sequence*** | ***(cDNA clone validation)*** |  |  |
| ZNF.1 | 5’-GCCCACAAAAAGAACAAGGA-3’ |  |  |
| ZNF.2 | 5’-GTCTGGCTGAAGGTGTCCTC-3’ |  |  |
| ZNF.3 | 5’-CCCACCCTCTACAACCTCAA-3’ |  |  |
| ZNF.4 | 5’-CACAGGGTAGGCATGGTTCT-3’ |  |  |
| ZNF.5 | 5’-AGAGCTGCGACAAGCAATTT-3’ |  |  |
| ZNF.6 | 5’-ACACAGGGTGCAGTGGTACA-3’ |  |  |
| ZNF.7 | 5’-GAACCCTGAGGCACCTAACA-3’ |  |  |
| ZNF.8 | 5’-GCACCTCCATGGTGTA-3’ |  |  |
| ZNF.9 | 5’-GCCAGATGACCTTCGAGAAC-3’ |  |  |
| ZNF.10 | 5’-GCCTTCCTCAATCATGTGGT-3’ |  |  |
